# How far is observation allowed in patients with ectopia lentis?

**DOI:** 10.1186/s40064-015-1239-5

**Published:** 2015-08-28

**Authors:** Toshihiko Matsuo

**Affiliations:** Department of Ophthalmology, Okayama University Medical School and Graduate School of Medicine, Dentistry, and Pharmaceutical Sciences, 2-5-1 Shikata-cho, Okayama City, 700-8558 Japan

**Keywords:** Ectopia lentis, Clinical decision, 25-gauge vitrectomy system, Lensectomy, Visual acuity, Marfan syndrome, Intraocular lens implantation, Observation, Aphakia, Children

## Abstract

Surgical timing for ectopia lentis has not been well described until now. The purpose of this study is to find a benchmark as to how far observation would be allowed in children with ectopia lentis when they and their families are reluctant to go through surgery. Retrospective review was made on 15 consecutive patients (14 children and one adult) with ectopia lentis in both eyes, seen at a referral-based institution in 5 years from April 2008 to March 2013, to survey the reasons for continuing observation or deciding surgical intervention. The diagnoses were Marfan syndrome in six patients, familial ectopia lentis in six, and sporadic ectopia lentis in three. Observation was continued in nine patients with the age at the final visit, ranging from 4 to 17 (median 9) years, because six children had good visual acuity at both near and distant viewing with glasses, and three children had visual acuity of 0.4 at near viewing despites poor visual acuity at distant viewing with glasses. In contrast, lensectomy was determined in six patients (5 children and one adult) with the age at surgery, ranging from 4 to 36 (median 9) years, and the age at the final visit, ranging from 7 to 42 (median 11) years, mainly because of poor visual acuity at near and distant viewing. More specific causes for surgeries in five children were the optical axis to become aphakic due to the progression of ectopia in the course in two children, lens dislocation to the anterior chamber after blunt eye injury in one child, and difficulty in studying at school classes in two children. One adult patient developed cataract in ectopic lenses. Lensectomy, combined with anterior vitrectomy, was done from two limbal side ports with a 25-gauge infusion cannula and vitreous cutter. Two patients at the age of 16 and 36 years, additionally, underwent intraocular lens-suturing in both eyes. In conclusions, observation was continued in children with ectopia lentis who had good visual acuity at near viewing. The visual acuity at near viewing, 0.4 or better, would give a benchmark for continuing observation in children with ectopia lentis.

## Background

Ectopia lentis is a congenital disorder in children to show malposition of the lens (Halpert and BenEzra 1996; Koenig and Mieler 1996). The center of the lens is not in alignment with the optical axis of the eyeball, and the equator of the lens with sparse ciliary zonules is visible in one or two quadrants under mydriasis and frequently even through the pupil in the normal size. In fact, the ectopic lens is hypoplastic by nature with its diameter smaller relative to the diameter of the ciliary body circumference, and thus, the lens appears to be located eccentrically. The small lens size in ectopia lentis is in marked contrast with the normal size of the lens in secondary lens subluxation, caused by blunt eye injury. Another conspicuous feature in ectopia lentis is lens coloboma with the decreased number of ciliary zonules.

Ectopia lentis occurs either in isolation or as a syndrome, including Marfan syndrome, Weill–Marchesani syndrome, and homocystinuria (Halpert and BenEzra 1996; Koenig and Mieler 1996). The isolated condition of ectopia lentis is either sporadic or familial, and the familial form usually shows an autosomal dominant trait. Genetic testing has been evolving in ectopia lentis (Chandra and Charteris 2014).

The extent of ectopia lentis and its change in the time course vary from patient to patient. Under the circumstances, there is no established standard of care, described in textbooks or the literature, as to how far observation would be allowed in children with ectopia lentis. Two apparent surgical indications for ectopia lentis at the textbook level are: (1) lens positioning with the lens edge bisecting the pupil, which makes impossible the optical correction of either the aphakic part or phakic part of the pupil, and (2) anterior displacement of the lens which causes secondary glaucoma.

In contrast, surgical methods for lensectomy have been well described in the literature. Pars plana lensectomy in both eyes, followed by aphakic correction with glasses or contact lenses, is a standard strategy for surgical intervention in children with ectopia lentis (Halpert and BenEzra 1996; Koenig and Mieler 1996; Anteby et al. 2003; Shortt et al. 2004; Konradsen et al. 2007; Kim et al. 2008; Babu et al. 2010). Trans-scleral suturing of the intraocular lens, concurrently with lensectomy, has been described in some patients with ectopia lentis (Omulecki et al. 1998; Ozdek et al. 2002).

In this study, I tried to find a benchmark as to how far observation was allowed in a consecutive series of 15 patients with ectopia lentis at one referral-based institution. In addition, a modified surgical method was described as to lensectomy, combined with anterior vitrectomy, using a 25-gauge infusion cannula and a vitreous cutter which were inserted from two side ports at the corneal limbus (Matsuo 2014).

## Results

The patients, involved in this study (Table 1), were 14 children, including 9 males and 5 females, in addition to one female adult (Case 3) who was the mother of two children (Cases 1 and 2). The diagnoses were Marfan syndrome in six patients, familial ectopia lentis in six, and sporadic ectopia lentis in three. Observation was chosen in nine patients with the age at the initial visit ranging from 2 to 9 (median 5) years and the age at the final visit ranging from 4 to 17 (median 9) years. In contrast, lensectomy was chosen in six patients with the age at the initial visit ranging from 3 to 36 (median 4) years, the age at surgery ranging from 4 to 36 (median 9) years, and the age at the final visit ranging from 7 to 42 (median 11) years. The patients were followed at the interval of 3, 4, or 6 months, based on their ages: younger children were more frequently seen while school-age children were seen mainly in school vacations.

**Table 1:** Summary of 15 consecutive patients with ectopia lentis

Case no/sex/age at initial visit/final visit (years)	Visible equator of the lens in both eyes	Family history of ectopia lentis	Diagnosis	Age at surgery	The reason for surgery	Visual acuity^a^ & refractive error (diopter) at distance RE LE	Visual acuity^a^ at near RE LE	Visual acuity^b^ & refractive error (diopter) at distance after surgery RE LE
1/M/3/16	Temporal	Grandfather (aphakia) Mother (Case 3) Sister (Case 2)	Familial ectopia lentis	16 IOL suture in both eyes	Difficulty in school class	0.07 s.-2.0 c.-2.0A180	0.6	0.6 s.+2.5 c.-5.0A10
						0.06 s.-2.0 c.-2.5A180	0.6	0.6 s.+0.75 c.-3.5A25
2/F/7/13	Temporal	Grandfather (aphakia) Mother (Case 3) Brother (Case 1)	Familial ectopia lentis	Not done		0.1 s.+2.0 c.-4.0A90	0.4	
						0.1 s.+2.0 c.-4.0A90	0.4	
3/F/36/42	Temporal	Father (aphakia) Son (Case 1) Daughter (Case 2)	Familial ectopia lentis	36 IOL suture in both eyes	Cataract in both eyes	0.1 s.-16.0 c.-1.0A180	0.1	1.0 s.-2.5 c.-0.5A125
						0.1 s.-16.0 c.-1.0A180	0.1	0.9 s.-0.75 c.-1.0A180
4/M/2/10	Nasal	Father (aphakia) Brother (Case 5) Sister (Case 6)	Marfan syndrome	Not done		1.0 s.-10.0 c.-2.0A180	0.9	
						0.4 s.-10.0 c.-3.0A10	0.3	
5/M/5/14	Nasal	Father (aphakia) Brother (Case 4) Sister (Case 6)	Marfan syndrome Funnel chest	12	Aphakic optical axis in RE	0.04 s.+14.5 c.-0.75A180	0.1	0.9 s.+15.0 c.-1.0A180
						0.8 s.-13.5 c.-3.0A20	0.6	1.5 s.+14.0 c.-1.5A180
6/F/9/17	Nasal	Father (aphakia) Brother (Case 4) Brother (Case 5)	Marfan syndrome Mitral valve prolapse	Not done		0.9 s.-8.5 c.-2.0A180	0.9	
						1.0 s.-8.5 c.-2.0A180	0.9	
7/M/6/15	Inferonasal	None	Marfan syndrome Scoliosis	Not done		0.9 s.-1.5	0.4	
						0.4 s.-1.25 c.-3.0A180	0.1	
8/F/2/8	Inferonasal	None	Marfan syndrome Mitral valve prolapse Scoliosis	Not done		0.5 s.-12.25	0.2	
						0.3 s.-12.25	0.4	
9/M/3/8	Temporal	None	Sporadic ectopia lentis	6	Traumatic lens dislocation to anterior chamber in RE	0.5 s.-8.0	0.5	1.0 s.+15.75 c.-2.0A180
						0.3 s.-8.0	0.3	1.0 s.+15.25 c.-1.25A180
10/M/3/7	Inferior	Mother (aphakia) Brother (Case 11)	Familial ectopia lentis	Not done		0.8 s.-3.0 c.-6.0A10	0.9	
						1.0 s.-0.5 c.-5.5A180	0.9	
11/M/2/4	Inferior	Mother (aphakia) Brother (Case 10)	Familial ectopia lentis	Not done		0.3 s.-4.5 c.-2.25A25	0.4	
						0.2 s.-7.0 c.-5.0A13	0.4	
12/F/5/9	Inferonasal	Father	Marfan syndrome	6	Difficulty in school class	0.2 s.-5.5 c.-5.0A50	0.3	1.2 s.+14.0 c.-1.0A180
						0.1 s.0 c.-4.0A180	0.1	1.5 s.+14.0 c.-1.5A180
13/M/4/7	Temporal	None	Sporadic ectopia lentis	4	Aphakic optical axis in RE	0.07 s-4.5 c.-2.75A165	0.2	1.0 s.+15.0 c.-1.0A20
						0.06 s.-7.0 c.-5.0A13	0.2	1.0 s.+15.0 c.-1.0A150
14/M/5/9	Inferonasal	Mother Grandmother	Familial ectopia lentis Moderate mental retardation Autism	Not done		0.1 s.-6.0	0.4	
						0.1 s.-6.0	0.4	
15/F/6/6	Inferior	None	Sporadic ectopia lentis	Not done		0.8 s.-0.5 c.-2.5A170	0.9	
						0.8 s.-1.0 c.-5.25A175	0.8	

*RE* right eye, *IOL* intraocular lens, *s*. spherical, *c.* cylindrical, *A* axis

^a^Visual acuity: best-corrected visual acuity at the final visit in patients with observation while best-corrected visual acuity just before the surgery in patients with lensectomy. Refractive error, determined basically by autorefraction and also by trial-lens exchange in elder patients

^b^Visual acuity: best-corrected visual acuity at the final visit

**Fig. 1:**
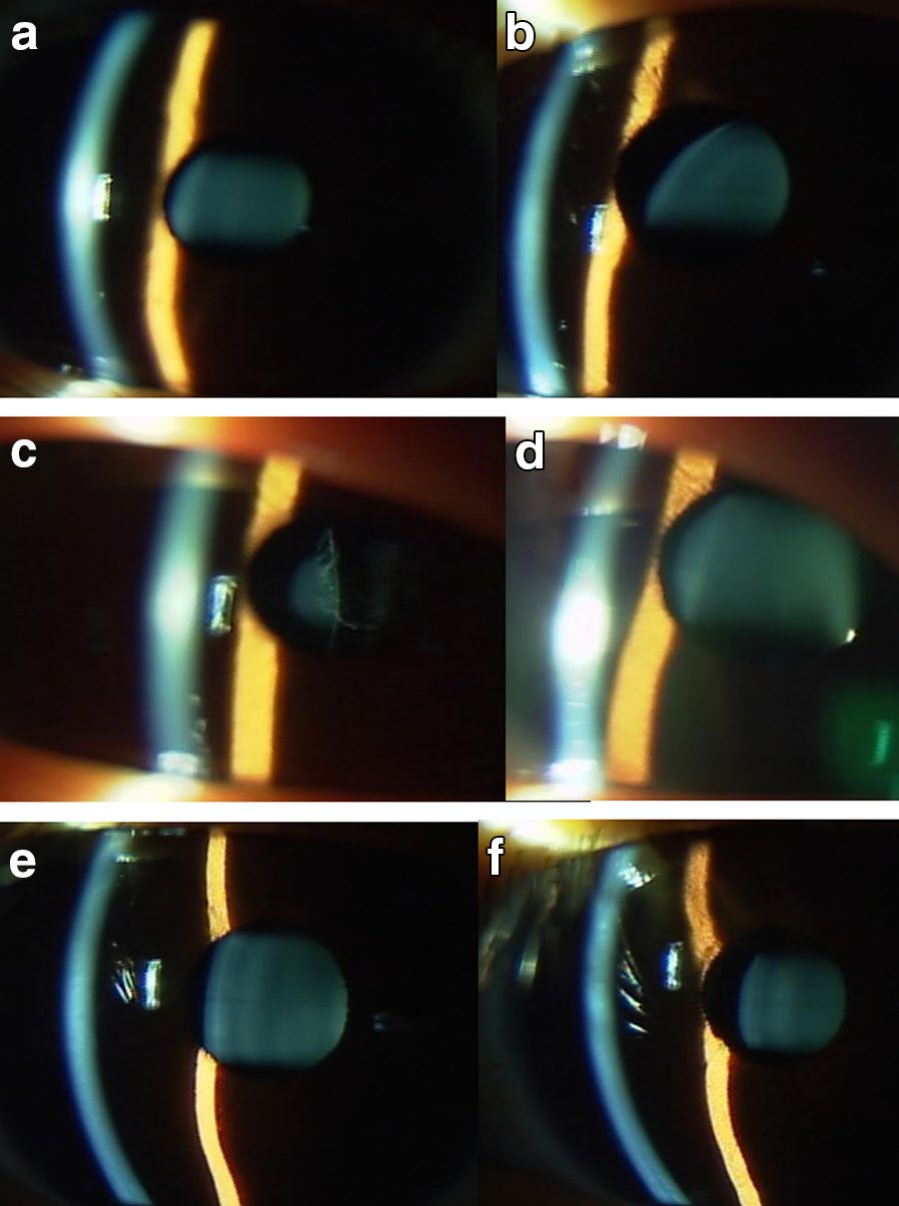
Case 4 (**a** right eye; **b** left eye), Case 5 (**c** right eye; **d** left eye), and Case 6 (**e** right eye; **f** left eye) with ectopia lentis in both eyes associated with Marfan syndrome. Brothers (Case 4 and Case 5) and a sister (Case 6) in a family with Marfan syndrome. Their father’s eyes are aphakic after lensectomy in both eyes for ectopia lentis with Marfan syndrome. Only Case 5 (**c** and **d**) underwent lensectomy in both eyes at the age of 12 years, due to aphakic optic axis in the right eye (**c**). Case 6 with smaller degrees of ectopia lentis in both eyes (**e** and **f**) at the age of 17 years still maintains good visual acuity at near and distant viewing while Case 4 (**a** and **b**) with ectopia lentis, more marked in the left eye (**b**), shows relatively worse visual acuity in the left eye at the age of 10 years

**Fig. 2:**
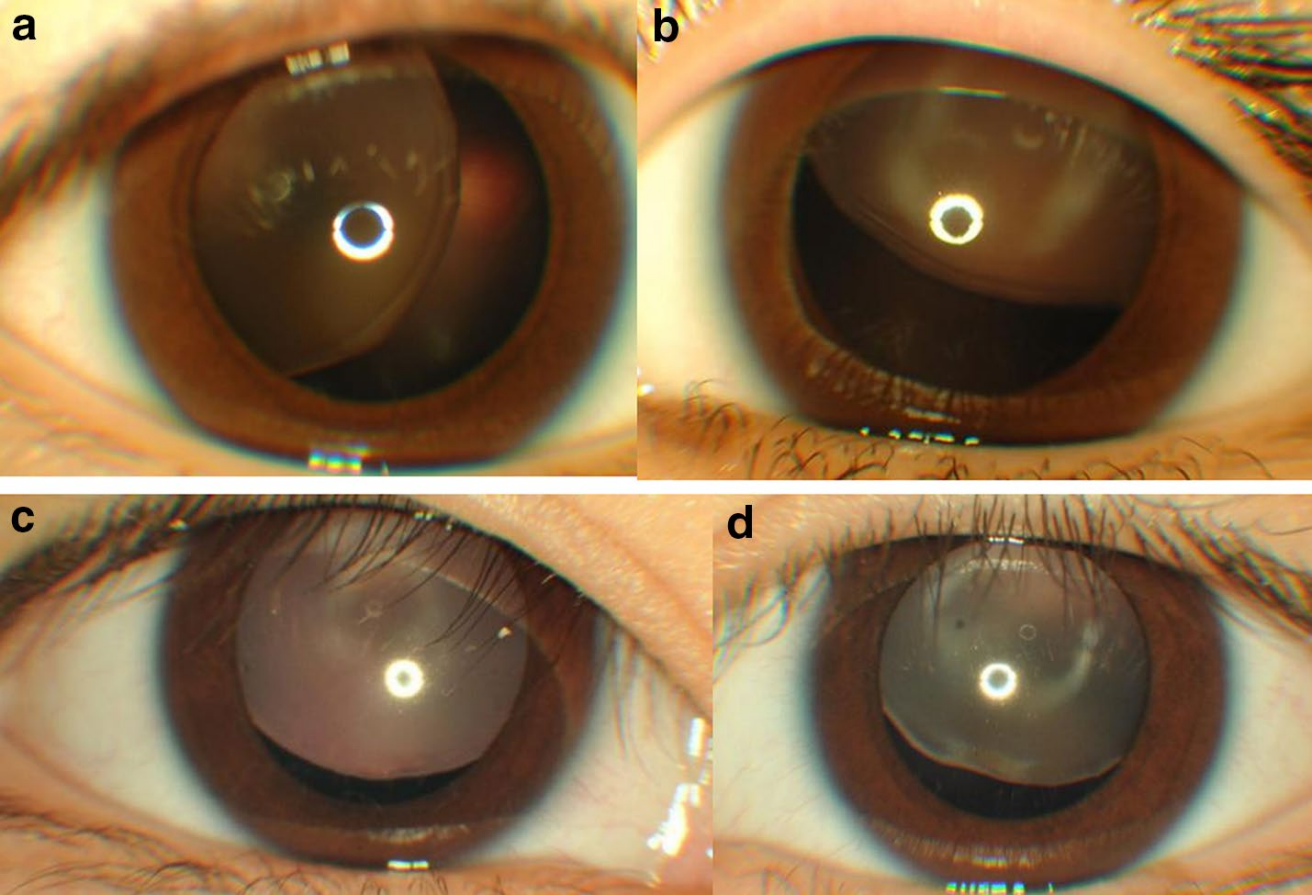
Case 12 (**a** right eye; **b** left eye) and Case 15 (**c** right eye; **d** left eye). Lensectomy in both eyes was done at the age of 6 years in Case 12 with ectopia lentis associated with Marfan syndrome, due to poor visual acuity at near and distant viewing. Her father is also diagnosed as Marfan syndrome. Case 15 with sporadic ectopia lentis in both eyes at the age of 6 years is followed due to good visual acuity at near and distant viewing

Observation in nine children was continued since six children (Figs. 1a, b, e, f, 2c, d) had good visual acuity at both near and distant viewing with glasses while three children had visual acuity of 0.4 at near viewing despites poor visual acuity of 0.1 or worse at distant viewing with glasses (Case 2, Case 11, and Case 14). All nine children showed regular astigmatism and had good compliance with spectacle wear. The best-corrected visual acuity between both eyes was basically at the same level in 7 of the 9 children with observation while the remaining two children (Case 4 and Case 7, Fig. 1a, b) showed rather marked difference in the visual acuity between both eyes. Occlusion therapy with a patch on the eye which had the better visual acuity, was not performed in these two children.

**Fig. 3:**
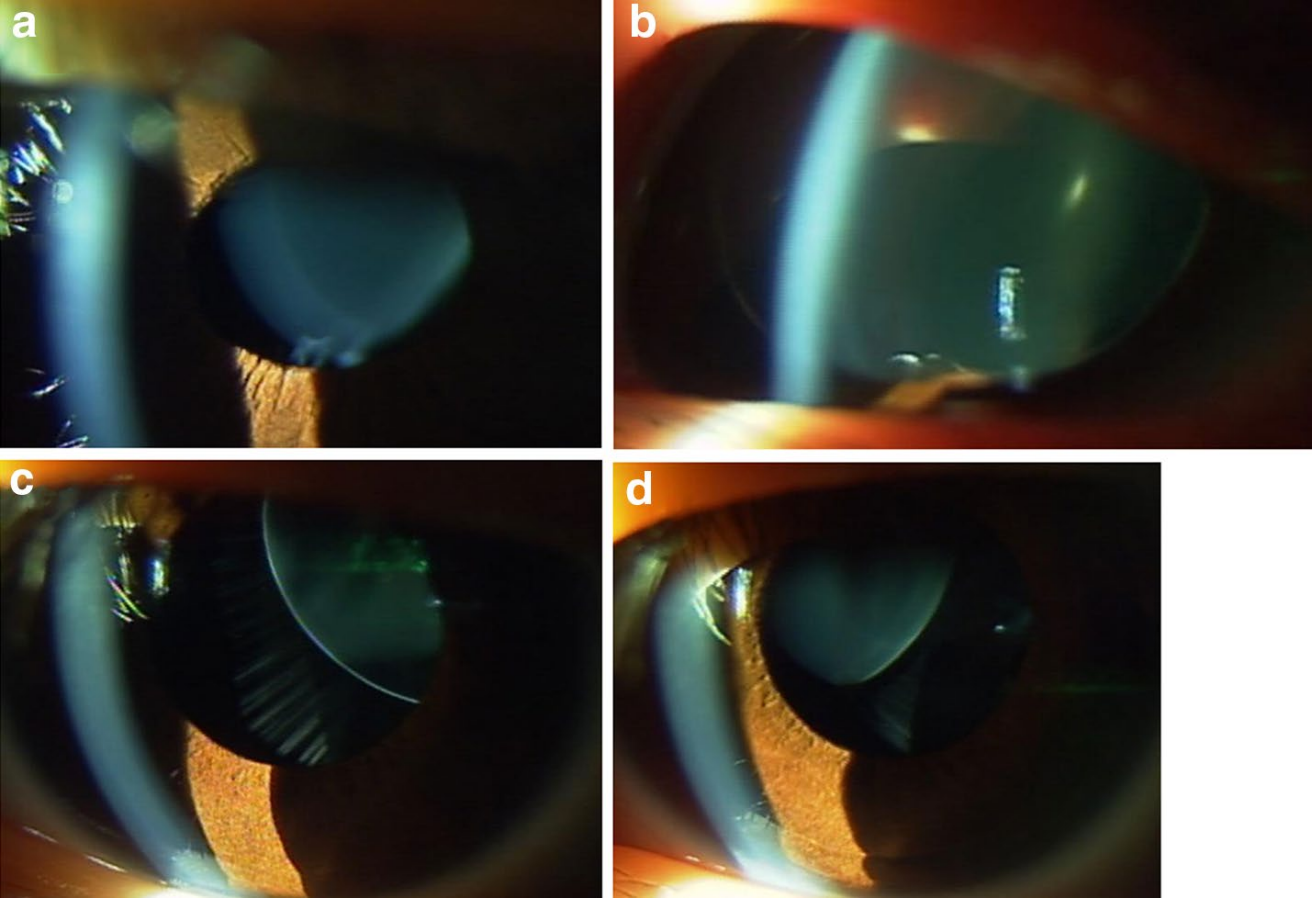
Case 9 (**a** before injury; **b** after blunt injury in right eye) and Case 13 (**c** right eye; **d** left eye) with sporadic ectopia lentis in both eyes. Case 9 underwent lensectomy in both eyes at the age of 6 years, due to traumatic dislocation of ectopic lens to the anterior chamber of the right eye (**b**). Case 13 underwent lensectomy in both eyes at the age of 4 years, due to aphakic optical axis in the right eye (**c**)

**Fig. 4:**
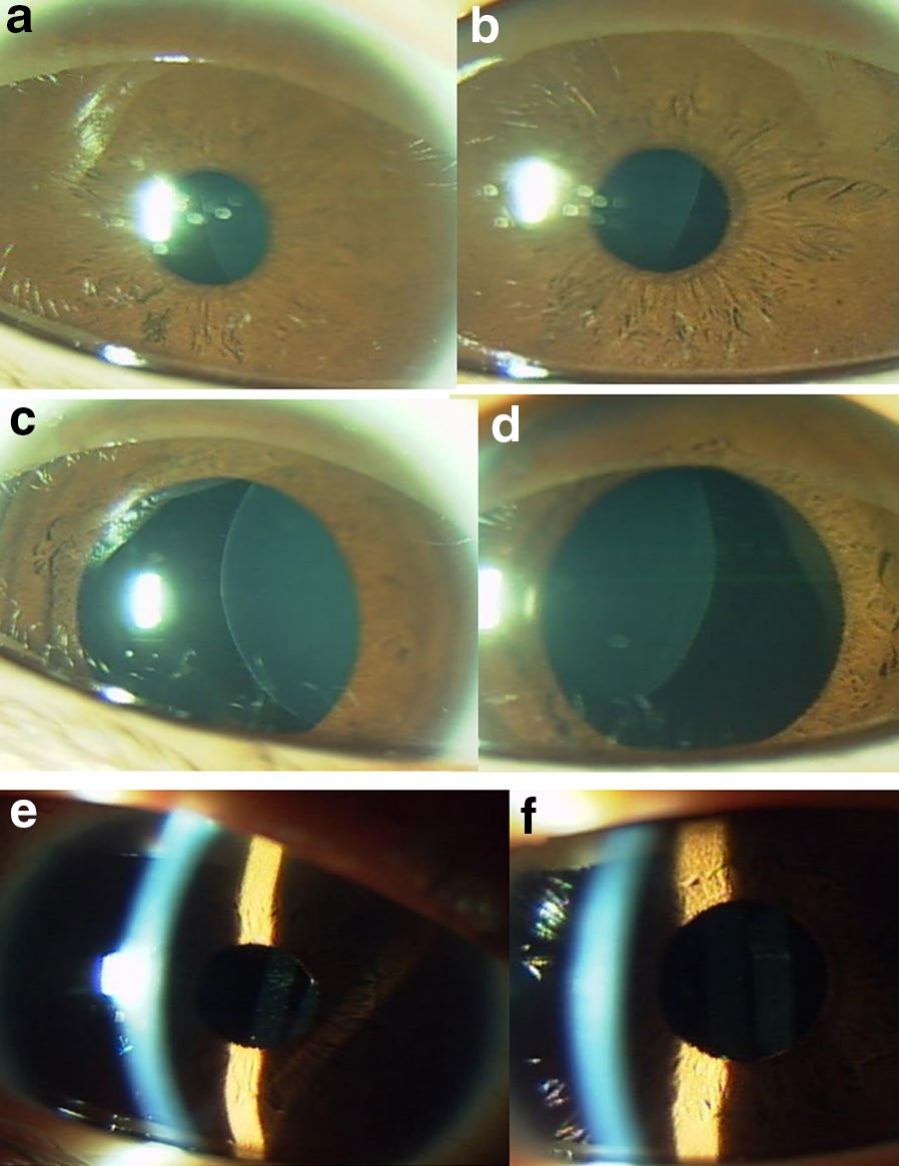
Case 1 (**a**, **c**, **e** right eye; **b**, **d**, **f** left eye) with familial ectopia lentis in both eyes. Ectopic lenses under the normal pupil (**a**, **b**) and under mydriasis (**c**, **d**), and intraocular lenses after lensectomy (**e**, **f**) at the age of 16 years. The mother (Case 3) also underwent lensectomy with intraocular lens-suturing at the age of 36 years while the sister (Case 2) with ectopia lentis at the age of 13 years is still followed due to good vision at near viewing

The main reason for lensectomy in the six patients was poor visual acuity at near and distant viewing. More specific causes for choosing surgery in the five children were the optical axis to become aphakic due to the progression of ectopia in the course in two children (Case 5 and Case 13, Figs. 1c, d, 3c, d), lens dislocation to the anterior chamber after blunt eye injury in one child (Case 9, Fig. 3a, b), and difficulty in studying at school classes in 2 children (Case 1: Fig. 4 and Case 12: Fig. 2a, b). One adult patient (Case 3) with ectopia lentis underwent lensectomy, combined with intraocular lens-suturing, because of deteriorated vision with cataract formation.

The best-corrected visual acuity after lensectomy only was 0.9 or better in both eyes of four children (Cases 5, 9, 12, and 13). In two patients with intraocular lens-suturing, a 16-year-old male (Case 1) obtained the best-corrected visual acuity of 0.6 in both eyes and his mother (Case 3) at the age of 36 years obtained the visual acuity of 0.9 or 1.0 in both eyes (Table 1). No patient showed any complications, including glaucoma and retinal detachment, after the surgery.

## Discussion

The goal of this study was to find a benchmark as to how far observation would be allowed in children with ectopia lentis when they and their families were reluctant to go through surgery. In other words, the aim was to elucidate which level of the visual acuity would be good enough to be followed in children with ectopia lentis. Most of cases with ectopia lentis are children who are in the process of growth of the ocular structure and development in the visual acuity. Therefore, clinical decision-making of either observation or surgical intervention is crucial in the follow-up of children with ectopia lentis from viewpoint of avoiding amblyopia.

In the present series of children, it is understandable that lensectomy is required in children who have developed the aphakic optical axis due to the deterioration of lens ectopia in the follow-up or who have abruptly developed lens dislocation to the anterior chamber after blunt ocular trauma. Even under the circumstances that unilateral lensectomy was indicated, lensectomy in both eyes was done in the children to seek postoperative corrections with glasses in the present series of children. In the study period, intraocular lens-suturing was not done in children at younger ages who required general anesthesia for surgery. In general, patients with significant ectopia lentis would be refracted with a high-grade plus lens to achieve better vision through the aphakic portion of the pupil. Bilateral lensectomy was chosen in this study since unilateral aphakic correction with glasses was inappropriate and intraocular lens-suturing was not adopted as a surgical method in children. There is also no choice but surgery in an adult patient who has developed cataract in ectopic lenses of both eyes.

Apart from these extreme cases, children with ectopia lentis have been followed to check the presence of the ectopic lens in the pupillary area and to measure the visual acuity and the intraocular pressure. The optical use of the lens periphery in ectopia lentis results in astigmatism with high-grade myopia. Glasses are prescribed, based on cycloplegic refraction with cyclopentolate, and the visual acuity was examined both at near viewing and at distant viewing. Full correction was not necessarily sought for a large degree of astigmatism or for high-grade myopia. Rather, emphasis was placed on the reading distance at near viewing when the glasses were prescribed.

The present study showed that children with ectopia lentis were observed with glasses as far as the visual acuity at the distant and near fixation was good enough for daily life and school learning. The threshold of the visual acuity for no problem in school learning is around 0.4–0.5 in decimals in the present series of children. In addition, the small part of children with ectopia lentis in this study showed better visual acuity only at near viewing, but not at distant viewing, leading to the observation. The clinical decision of observation was based, not only on daily attitude and activity of children at home and school, but also on wishes and the way of thinking of their parents. One timing for surgical intervention was around the entrance to elementary schools at the age of 6 years since good vision at distant viewing is required by schools to determine whether additional help is necessary or not after the admission. In this sense, a 4-year-old boy (Case 11), with distant visual acuity of 0.3 and near visual acuity of 0.4 at the final visit of this study, would choose surgery in coming years before the admission to an elementary school.

The main aim of surgical intervention in children with ectopia lentis is to regain good visual acuity at near and distant viewing and also to avoid or reverse the amblyogenic situation. In this study, the four children with lensectomy only, for ectopia lentis, showed good visual acuity with glasses in both eyes in the follow-up after the surgery. Immediately after the surgery, some children did show the difference in the best-corrected visual acuity between the right eye and left eye, suggesting the presence of unilateral amblyopia. They, in turn, gained the good visual acuity with glasses in both eyes in the short-term course of the follow-up, suggesting also that amblyopia, if ever exist, would be shallow.

Furthermore, it should be noted that a 36-year-old patient (Case 3) with ectopia lentis gained the visual acuity around 1.0 in both eyes after lensectomy, combined with intraocular lens-suturing. She postponed lensectomy to the timing only after the development of cataract in adulthood since her father, also with ectopia lentis, underwent intracapsular cataract extraction later in the life. She passed the vision test for drivers’ license after the surgery. She might have undergone surgery earlier in the life as did her son (Case 1) if the modern technique of lensectomy, as described in this study, would have been available.

The surgical outcomes in the present series of patients, altogether, indicate that the children and the adult with ectopia lentis who underwent the surgery did not have amblyopia despites their limited levels of best-corrected visual acuity before the surgery. The strategy for observation in the other children in this study would be supported from the non-amblyogenic point of view in ectopia lentis. Two children (Case 4 and Case 7) with observation at the age of 10 and 15 years, respectively, at the final visit in the present study, did show large interocular difference in the visual acuity. They did not have difficulty in school classes, and thus, did not choose surgery. The difference in best-corrected visual acuity between both eyes in these two patients would result from the different extent of ectopia, and would not necessarily mean the presence of amblyopia. Nonetheless, amblyopia in one eye would not be ruled out in these two patients.

This study does not advocate a reluctance to do surgery in children with ectopic lentis because of a risk of glaucoma or retinal detachment. Or rather, the surgical indication should be determined carefully on the balance of surgical risks versus benefit in daily life and school learning, in the conversation with patients and their families. The assessment of visual acuity at near viewing and distant viewing, together with appropriate prescription of glasses, is mandatory at repeat visits in the follow-up. The study was based on one surgeon’s experience in managing a small number of patients with ectopia lentis, and thus, the results would not necessarily be generalized to the standard of care for managing ectopia lentis. With the limitation of the study in mind, the clinical benchmark, found as one surgeon’s perspective, might be useful, especially when surgeons and patients’ families would waver in choice of observation or surgical intervention.

## Conclusions

This is the first study, to the best of my knowledge, to show the strategy for observation or surgical intervention in children with ectopia lentis. The visual acuity at near viewing is one key for determining the choice of observation or surgical intervention. Poor levels of best-corrected visual acuity, less than 0.4, both at near viewing and at distant viewing, would not necessarily mean the presence of amblyopia in children with ectopia lentis, as evidenced by children who underwent surgery in this study.

Advances in lensectomy from limbal side ports with a 25-gauge infusion cannula and vitreous cutter (Matsuo 2014), as described in this study, might lead to easy choice of surgical intervention in children with ectopia lentis. Lensectomy at younger age naturally leads to the loss of accommodation, and would have still a risk, though at a low level, for developing complications, such as glaucoma and retinal detachment. Observation would be allowed in children with ectopia lentis, having the visual acuity of at least 0.4 at near viewing, when they and their families are reluctant to go through surgery.

## Methods

### Patients

Retrospective review was made on medical records of 15 consecutive patients with ectopia lentis in both eyes, seen at Okayama University Hospital in 5 years from April 2008 to March 2013. This study adhered to the tenets of the Declaration of Helsinki and was approved by the institutional review board (Ethics Committee of Okayama University Graduate School of Medicine, Dentistry, and Pharmaceutical Sciences) as a retrospective case-series study. The reasons why observation or lensectomy was chosen were surveyed on the medical records.

### Surgical methods

Lensectomy, combined with anterior vitrectomy, was done from two side ports at the corneal limbus with a 25-gauge infusion cannula and vitreous cutter (ACCURUS Surgical System and CONSTELLATION Vision System, Alcon, Fort Worth, TX, USA) (Matsuo 2014). Dissection was made on the lens capsule with a 20-gauge knife (Corneal/Scleral V-LANCE Knife, Alcon), inserted through the limbal side port. Under the continuous irrigation with a 25-gauge infusion cannula held at one side port, the lens cortex and nucleus were aspirated inside the capsular bag by a 25-gauge vitreous cutter, inserted from the other side port, in a changing mode for either cutting or aspiration. Finally, the lens capsule with the residual cortex was cut with a cutter. At the equatorial area of the lens in quadrants with ciliary zonules in the normal density, the lens capsule was cut under its visualization by scleral depression.

Two patients at the age of 16 and 36 years underwent intraocular lens-suturing in both eyes. In these patients, conjunctival incision was made along the corneoscleral limbus in the upper half circumference. Three 25-gauge trocars were inserted obliquely at the pars plana, 3 mm from the limbus, with one trocar for an infusion cannula through the displaced conjunctiva and then through the sclera in the inferotemporal quadrant, and with the other two trocars directly through the bared sclera. A corneoscleral tunnel with 7-mm width was made and an intraocular lens (CZ70BD, Alcon) with 10-0 polypropylene sutures (PC-9, Alcon), tightened at the end-ring of both haptics, was inserted. The strings were beforehand sutured through the sclera 3 mm from the limbus and the intraocular lens was fixed in the posterior chamber. Vitrectomy was done through the trocars placed in the pars plana. These two patients underwent surgery under local anesthesia while the remaining four patients at younger ages underwent surgery under general anesthesia. All surgeries were done by a single ophthalmologist (T.M.).

### Visual acuity testing

The visual acuity at distant viewing was tested at the distance of 5 m with internationally standard Landolt-C charts. Children at younger ages were tested with Landolt-C cards at 2.5 m for distant viewing and the visual acuity at 2.5 m was converted to that presumed at the measuring distance of 5 m (Matsuo 2014). The visual acuity at near viewing was tested at 30 cm with a hand-held Landolt-C cards for near viewing. The intraocular pressure in children was measured with a hand-held tonometer (Icare TA01i, Icare Finland, Helsinki). The other standard ophthalmological examinations included table-fixed pneumatic tonometry, hand-held or table-fixed autorefraction, hand-held or table-fixed slit-lamp biomicroscopy, and funduscopy.
